# Methane breath tests and blood sugar tests in children with suspected carbohydrate malabsorption

**DOI:** 10.1038/s41598-020-75987-6

**Published:** 2020-11-04

**Authors:** Christof Schneider, Klaus D. Wutzke, Jan Däbritz

**Affiliations:** 1 Department of Pediatrics, Rostock University Medical Center, Ernst-Heydemann-Str. 8, 18057 Rostock, Germany; 2 Centre for Immunobiology, Blizard Institute, Barts Cancer Institute, The Barts & The London School of Medicine & Dentistry, Queen Mary University of London, London, UK

**Keywords:** Paediatric research, Gastroenterology, Malnutrition

## Abstract

Carbohydrate malabsorption and subsequent gastrointestinal symptoms are a common clinical problem in pediatrics. Hydrogen (H_2_) and methane (CH_4_) breath tests are a cheap and non-invasive procedure for diagnosing fructose and lactose malabsorption (FM/LM) but test accuracy and reliability as well as the impact of non-hydrogen producers (NHP) is unclear. CH_4_ breath tests (MBT), blood sugar tests (BST) and clinical symptoms were compared with H_2_ breath tests (HBT) for FM/LM. 187/82 tests were performed in children (2 to 18 years) with unclear chronic/recurrent abdominal pain and suspected FM/LM. In FM and LM, we found a significant correlation between HBT and MBT/BST. In LM, MBT differentiated most of the patients correctly and BST might be used as an exclusion test. However, additional MBT and BST had no diagnostic advantage in FM. NHP still remain a group of patients, which cannot be identified using the recommended CH_4_ cut-off values in FM or LM. Reported symptoms during breath tests are not a reliable method to diagnose FM/LM. Overall a combined test approach might help in diagnosing children with suspected carbohydrate malabsorption.

## Introduction

Carbohydrate intolerance/malabsorption is often suspected in pediatric patients with food intake related chronic abdominal pain. However, clinical manifestation of carbohydrate intolerance/malabsorption in children varies and unspecific symptoms range from recurrent abdominal pain (RAP), bloating and nausea to flatulence and diarrhea^1–3^. Because similar symptoms can be caused by other more severe gastrointestinal diseases it is important to establish a correct diagnosis. In this study we focused on breath tests, which have a special role in diagnosing carbohydrate malabsorption and other gastrointestinal disorders in children non-invasively. However, the diagnostic reliability of breath tests in children with suspected carbohydrate malabsorption including defined cut-off levels, sensitivity and specificity is still unclear and a worldwide consensus does not exist^4,5^. For example, it has been shown that the induction of symptoms following sugar ingestion (carbohydrate intolerances) appears to be more relevant than carbohydrate malabsorption (positive breath test) in functional gastrointestinal (GI) diseases^6^. GI symptoms rather correlate with discomfort/intolerance during breath tests than with malabsorption. For example, a lack of association of the induction of symptoms with the presence of malabsorption of fructose has been shown^7^. Due to the relatively low reliability of breath tests and the missing correlation of GI symptoms and malabsorption has questioned the usefulness of performing breath test in patients with suspected carbohydrate malabsorption. Instead, the use of symptom-based provocation test before dietary interventions in functional GI diseases has been suggested^8^.

Fructose is increasingly used as a sweetener and added to processed food, for example fruit juices, other beverages and candy as well. Fructose malabsorption (FM) is common, it affects approximately 5% of the population^9^. It is a monosaccharide which is absorbed by carrier-mediated facilitated diffusion via the GLUT5 transporter across the intestinal apical membrane and then transported from the cytosol to the blood by the basolateral transporter GLUT2^10,11^. Based in the increased fructose intake it has been proposed that other transporters in addition to GLUT5, e. g. GLUT2, might be involved during the transport of fructose via the apical cell membranes in the small intestine^12^. A deletion of GLUT 5 in mice can reduce the fructose intake significantly and, therefore, cause symptoms of carbohydrate malabsorption^13^. However, fructose malabsorption is not associated with intestinal GLUT5 and GLUT2 expression in human adults^12^. Furthermore, the simultaneous consumption of glucose can increase fructose absorption and prevent GI symptoms. The reason for this absorption enhancing effect is not fully understood. Fructose could be passively absorbed due to water absorption induced by glucose. Another explanation might be a glucose dependent fructose co-transport system. An increase of GLUT2 in the apical membrane after glucose ingestion was observed in rats which suggests an involvement of the fructose and glucose transporter GLUT2^14^. Thus, it is in fact the fructose in excess of glucose that may be malabsorbed, as opposed to total fructose. A high intake of fructose may result in symptoms like nonspecific diarrhea, intestinal gas and recurrent abdominal pain^1^. Intolerance to foods containing lactose is common^15^.

The prevalence of lactose malabsorption (LM) in Germany is 16% and has an overall frequency of around two-thirds of the world’s population^16^. Lactose is a non-absorbable disaccharide which is normally hydrolyzed by lactase into glucose and galactose^2^. A reduced expression or impaired enzyme activity can lead to LM^17^.

Carbohydrates which are not absorbed in the small intestines are fermented by anaerobic colonic bacteria. This process results in the formation of gas (hydrogen [H_2_]_,_ methane [CH_4_], carbon dioxide [CO_2_]) and short fatty acids by the anaerobic bacterial flora^18^. Fractions of H_2_ and CH_4_, which are not produced by human cells, diffuse passively into blood capillaries and are eliminated by breath where it can be measured by gas chromatography^19^.

There is a small percentage of patients which have an insufficient increase in breath H_2_ resulting in a negative H_2_ breath test (HBT). It is caused by their colonic flora which is unable to produce H_2_ during fermentation. Patients having carbohydrate malabsorption and a negative HBT result are called non-hydrogen-producers (NHP). The reported prevalence of NHP varies widely from 2 to 43%^4^. Therefore, breath CH_4_ might represent an alternative gaseous marker for this subgroup^4,20^. However, children who are suffering from carbohydrate malabsorption, can also show a negative CH_4_ breath test (MBT) even though the HBT is positive. These patients are called non-methane-producer (NMP). The absence of CH_4_ during MBT does not necessarily mean that the methanogenic flora is absent. Mathur et al. showed that methanogens are present in the majority of healthy adults’ colonic flora^21^. In other studies, the rate of CH_4_ producers in healthy subjects varies from 30 to 35%^22–24^. Another potential explanation for the discrepancy between carbohydrate intolerance and carbohydrate malabsorption besides NHP/NMP is an alternative mechanism for symptom generation.

BST might represent a (more invasive) alternative to HBT/MBT for diagnosing FM/LM. An abnormal BST indicates the absence of an increase in blood glucose levels after the ingestion of fructose/lactose due to a lack of absorption (FM) or hydrolyzation (LM)^25^.

The aim of our study was to determine the validity and reliability of MBT and blood sugar tests (BST) in pediatric patients with suspected carbohydrate malabsorption. We compared and combined HBT and MBT, BST and clinical symptoms in order to establish reliable cut-off values and a diagnostic algorithm for diagnosing LM and FM in children.

## Patients and methods

### Patients

In this retrospective single-center study we evaluated the diagnostic value of HBT and MBT in pediatric patients with unclear chronic abdominal pain and suspected LM and/or FM. Expiratory H_2_ and CH_4_ concentrations were correlated with simultaneously measured blood sugar levels and clinical/gastrointestinal symptoms. Some patients were tested for LM and FM and had, therefore, more than one breath test but only on different days. Patients underwent routine follow-up investigations if the tests were negative and symptoms persisted. We included patients with unclear chronic/recurrent abdominal pain, which were referred to the Department of Pediatrics at the Rostock University Medical Center (Rostock, Germany) by pediatricians between January 2012 and May 2015. Each patient underwent a clinical evaluation by one of our physicians and was monitored during the tests by a trained nurse. Clinical evaluation included a detailed medical history, a physical examination and blood tests if indicated. Parents were informed about the pre-test requirements prior to the test. If the tests were positive, all patients received an immediate dietary counselling by a pediatrician and in addition they were offered an appointment for further dietary counselling by a dietician at our hospital. Since substantial overlap between carbohydrate malabsorption and other gastroenterological diseases has been observed^26^, patients with known gastroenterological diseases (including hereditary fructose intolerance but except carbohydrate malabsorption) were excluded from the study in both groups (FM, LM).

### Pre-test conditions

Patients had to discontinue antibiotics, probiotics, prokinetic agents, laxatives or fluids for bowel/colonoscopy preparation 4 weeks before the breath test. Three days prior to the test fiber rich, highly saccharated or bloating food had to be avoided. Patients had to fast before the test for 8–12 h depending on their age. Patients were not allowed to eat or drink during the tests. Sport, smoking, chewing gum and brushing their teeth was also forbidden during the test. Compliance to the pre-test procedures was assessed by a checklist prior to the test. Patients were excluded from the study if the pre-test conditions were not followed.

### Breath tests

Breath tests were performed in our outpatient department in the morning and supervised by a trained nurse. Measures were taken at baseline and during the test at 30 min, 60 min, 90 min, 120 min, 150 min and 180 min. An overview of the test procedure is outlined in Table 1. The test solution consisted of 1 g fructose per kilogram bodyweight (max. 25 g) dissolved in water (10% solution) or 2 g lactose per kilogram bodyweight (max. 50 g) dissolved in water (20% solution). H_2_ and CH_4_ levels of end-expiratory breath samples were analyzed within 24 h after collections on a stationary non invasive breath/trace gas analyzer “Breath Tracker SC” (QuinTron, Milwaukee, USA) and recorded by a trained technician. Alveolar air was collected using “Alveo Sampler” (QuinTron, Milwaukee, USA), a mouthpiece connected via a Y-piece to a polyethylene bag and a syringe. After deep inspiration followed by a brief apnea period, patients exhaled into the polyethylene bags and only end-expiratory air after a 10 s expiration was collected. The polyethylene bags were sealed immediately after collecting each breath sample. Each sample was measured twice and mean values of these two measurements were used for calculating delta values. The threshold for detecting H_2_ and CH_4_ with our stationary gas chromatograph is 1 ppm, with an accuracy of ± 3 ppm of full scale.

**Table 1:** Overview of test procedures.

Time, minutes	0	15	30	45	60	90	120	150	180
Blood sugar test (BST)	X	X	X	X	X				
Hydrogen (H_2_) breath test (HBT)	X		X		X	X	X	X	X
Methane (CH_4_) breath test (MBT)	X		X		X	X	X	X	X
Symptoms questionnaire	X		X		X	X	X	X	X

We considered an increase of breath H_2_ ≥ 20 ppm in at least one measurement as positive^4,27,28^. For breath CH_4_ we considered a CH_4_ concentration ≥ 10 ppm at any measurement as a positive result^28^ and compared it with a lower threshold of ≥ 5 ppm and a threshold calculated by receiver operating characteristic (ROC) curve analyses. Patients having an elevated baseline value (0 min: H_2_ > 20 ppm/CH_4_ ≥ 10 ppm) were excluded. Patients with LM/FM having a baseline breath CH_4_ > 3 ppm were defined as CH_4_ producers. A patient with a positive HBT result was considered having FM or LM, respectively. These results were used as “gold standard” to calculate sensitivity, specificity, positive predictive values (PPV) and negative predictive values (NPV) for MBT, BST and symptoms^27^.

### Blood sugar tests

Blood sugar levels were measured using a handheld device called “ACCU-CHEK Inform II” (Roche, Basel, Switzerland). Blood samples were obtained from a clean and dry fingertip after skin disinfection. Immediately after pricking the fingertip with a lancet, the blood drop was applied to the test strip. An increase of blood glucose < 1.1 mmol/L during all BST was considered positive indicating carbohydrate malabsorption^2,28^. Delta values were calculated using the blood sugar level at a certain time (e.g., 15 or 30 min) and then being subtracted from the baseline value (0 min).

### Symptoms and subject conditions

New onset of (gastrointestinal) symptoms during the breath tests was documented based on a symptoms questionnaire before the test at baseline and during the test at different time points (30 min, 60 min, 90 min, 120 min, 150 min, 180 min). The questionnaire covered the following symptoms (yes/no answers): abdominal pain, bloating, nausea, flatulence, vomiting, diarrhea. The test would have been stopped if a patient had not tolerated the test conditions. All breath test results were interpreted by an experienced pediatric gastroenterologist who was blinded to symptoms and subject conditions.

### Data analysis

We used Microsoft Excel Version 16.26 for Mac (Microsoft Corporation, Redmond, USA) for data collection. SPSS Version 25 for Windows (IBM Corporation, Armonk, USA) was used for statistical analysis and the graphs were produced in Sigma Plot 13.0 (Systat Software GmbH, Germany). Percentages are provided for categorical variables. Continuous variables are expressed as median and range. ROC curve analyses were used to determine the statistically optimal cut-off value for breath tests based on sensitivity and specificity^29^. Cohen's κ was used to determine if there was an agreement between HBT and MBT/BST/symptoms during the breath tests. Standard error (SE), 95% confidence intervals (CI) were calculated and provided. Pearson X^2^-Test and Fisher’s exact test were used to verify if two or more categorial variables are statistically dependent.

### Ethics

The study was carried out in accordance with the declaration of Helsinki’s ethical principles for medical research involving human subjects and approved by the ethics committee of the University Medical Center Rostock, Germany (reference no. A 2015–0091). Informed consent was obtained from all subjects or, since most subjects were under 18, from a parent and/or legal guardian.

## Results

### Patients

A total of 269 breath tests were performed on 238 children aged from 2 to 18 years (mean age of patients with suspected FM: 11.0 years and 10.4 years in patients with suspected LM) according to our study protocol (Table 1). Detailed patients’ characteristics are provided in Table 2. Diagnostic tests were performed in 187 patients with suspected FM, while 82 patients were tested for LM. Out of 238 patients, 31 children (13%) were tested for both types of carbohydrate malabsorption with a median time interval of 14 days between the two breath tests. Based on the test protocols, none of the tests had to be terminated due to patients’ intolerance of the test conditions. Patients having an elevated baseline value (0 min: H_2_ > 20 ppm/CH_4_ ≥ 10 ppm) were excluded. This was the case in suspected FM for 51 patients (30 with an elevated H_2_ baseline, 14 with an elevated CH_4_ baseline and 7 with elevated baseline values in H_2_ and CH_4_). In suspected LM, we excluded 16 patients (11 with an elevated H_2_ baseline, 4 with an elevated CH_4_ baseline and 1 with elevated baseline values in H_2_ and CH_4_).

**Table 2:** Characteristics of patients with suspected carbohydrate malabsorption and overview of test results.

Patients	All	2–5 years	6–8 years	9–11 years	12–14 years	15–18 years
**Tests, n (%)**						
Fructose	187 (100)	27 (14)	35 (19)	47 (25)	35 (19)	43 (23)
Lactose	82 (100)	20 (24)	13 (16)	17 (21)	14 (17)	18 (22)
**Sex, female/male ratio**						
Fructose	1.9	2.0	1.5	1.8	0.8	3.3
Lactose	1.8	1.5	0.6	2.4	1.8	2.6
**Age, years (range**/**mean)**						
Fructose	2.3–18 (11.0)	4.5	7.7	10.4	13.3	16.4
Lactose	2.9–18 (10.4)	4.5	7.1	10.7	13.6	16.5
**HBT positive, n (%)**						
Fructose	113 (60)	23 (12)	24 (13)	32 (17)	20 (11)	14 (7)
Lactose	16 (20)	3 (4)	3 (4)	2 (2)	3 (4)	5 (6)
**MBT positive, n (%)**						
Fructose	66 (35)	16 (9)	19 (10)	20 (11)	7 (4)	4 (2)
Lactose	14 (17)	2 (2)	3 (4)	2 (2)	4 (5)	3 (4)
**BST positive, n (%)**						
Fructose	109 (58)	13 (7)	18 (10)	27 (14)	17 (9)	34 (18)
Lactose	12 (15)	0 (0)	1 (1)	3 (4)	3 (4)	5 (6)
**Symptoms positive, n (%)**						
Fructose	51 (27)	11 (6)	5 (3)	13 (7)	6 (3)	16 (9)
Lactose	23 (28)	2 (2)	2 (2)	7 (9)	4 (5)	8 (10)
**NMP positive, n (%)**						
Fructose	47 (25)	7 (4)	5 (3)	12 (6)	13 (7)	10 (5)
Lactose	3 (4)	1 (1)	0 (0)	0 (0)	0 (0)	2 (2)

*HBT* hydrogen breath test (an increase of H_2_ ≥ 20 ppm over baseline was considered positive). *MBT* methane breath test (a CH_4_ concentration ≥ 10 ppm at any time point was considered positive). *BST* Blood sugar test (an increase of blood glucose < 1.1 mmol/L at any time point was considered positive). *Symptoms* patients who showed or reported any symptoms during the test procedure. *NMP* non-methane producer (patients with a positive HBT but with a negative MBT).

### Breath test results

Following the cut-off values recommended by the “North American Consensus Conference for Hydrogen and Methane Breath Testing in Gastrointestinal Disorders” (NACC)^27^, FM was diagnosed by HBT in 113 (60%) patients whilst 16 (20%) had a positive HBT for LM (increase in H_2_ concentration ≥ 20 ppm above baseline). Increased CH_4_ levels ≥ 10 ppm during MBT were seen in 66 (35%)/14 (17%) patients with suspected FM/LM. For FM/LM, a negative MBT in combination with a positive HBT (non-methane producer; “false negative”) was found in 47 of 113 (42%)/3 of 16 (19%) patients. MBT for diagnosing FM using current cut-off values had a sensitivity of 58% and a specificity of 100% (Table 3). MBT had a sensitivity of 81% and a specificity of 99% in patients with suspected LM. PPV and NPV values are provided in Table 4.

**Table 3:** Comparison of methane breath tests, blood sugar tests and symptoms with results of hydrogen breath tests in patients with suspected fructose malabsorption.

	HBT (n = 187)		Sensitivity (%)	Specificity (%)	PPV (%)	NPV (%)
	Positive, n (%)	Negative, n (%)				
**MBT**						
Positive	66 (35)	0 (0)	58	100	100	61
Negative	47 (25)	74 (40)				
**BST**						
Positive	59 (32)	50 (27)	52	32	54	31
Negative	54 (29)	24 (13)				
**Symptoms**						
Positive	31 (17)	20 (11)	27	73	61	40
Negative	82 (44)	54 (29)				

*HBT* hydrogen breath test (an increase of H_2_ ≥ 20 ppm over baseline was considered positive). *MBT* methane breath test (a CH_4_ concentration ≥ 10 ppm at any time point was considered positive). *BST* blood sugar test (an increase of blood glucose < 1.1 mmol/L at any time point was considered positive). *Symptoms* patients who showed or reported any symptoms during the test procedure. *PPV* positive predictive value. *NPV* negative predictive value.

**Table 4:** Comparison of methane breath tests, blood sugar tests and symptoms with results of hydrogen breath tests in patients with suspected lactose malabsorption.

	HBT (n = 82)		Sensitivity (%)	Specificity (%)	PPV (%)	NPV (%)
	Positive, n (%)	Negative, n (%)				
**MBT**						
Positive	13 (16)	1 (1)	81	99	93	96
Negative	3 (4)	65 (79)				
**BST**						
Positive	8 (10)	4 (5)	50	94	67	89
Negative	8 (10)	62 (76)				
**Symptoms**						
Positive	8 (10)	15 (18)	50	77	35	86
Negative	8 (10)	51 (62)				

*HBT* hydrogen breath test (an increase of H_2_ ≥ 20 ppm over baseline was considered positive). *MBT* methane breath test (a CH_4_ concentration ≥ 10 ppm at any time point was considered positive). *BST* blood sugar test (an increase of blood glucose < 1.1 mmol/L at any time point was considered positive). *Symptoms* patients who showed or reported any symptoms during the test procedure. *PPV* positive predictive value. *NPV* negative predictive value.

There was a significant correlation between MBT and HBT in FM (Pearson-X^2^-Test: p < 0.001; Table 5), whereas the relative strength of the relationship was medium (Phi Coefficient: φ = 0.598, p < 0.001). Cohen's κ was performed to determine if there is an agreement between MBT and HBT in FM^30,31^ and showed a moderate agreement (κ = 0.526; 95% CI 0.422–0.630; p < 0.001). Breath tests in patients with suspected LM also had a significant correlation between MBT and HBT (Fisher’s exact test, p < 0.001). The relative strength of this relationship was strong (Phi Coefficient: φ = 0.84, p < 0.001) and Cohen’s κ indicated an excellent agreement (κ = 0.837; 95% CI 0.682–0.992; p < 0.001)^30,31^.

**Table 5:** Correlation of methane breath tests, blood sugar tests and symptoms in carbohydrate malabsorption.

	Independence				Association		Reliability				
	Pearson’s Chi-Squared test	Fisher’s exact test	df	P	Phi (φ)	P	Kappa (κ)	P	SE	95% CI_low	95% CI_high
**HBT fructose**											
MBT	66.796	–	1	0.000	0.598	0.000	0.526	0.000	0.053	0.422	0.630
BST	4.337	–	1	0.037	− 0.152	0.037	− 0.152	0.037	0.071	− 0.291	− 0.013
Symptoms	0.004	–	1	0.951	0.040	0.951	0.004	0.951	0.058	− 0.110	0.118
**HBT Lactose**											
MBT	–	57.828	1	0.000	0.840	0.000	0.837	0.000	0.079	0.682	0.992
BST	–	19.902	1	0.000	0.493	0.000	0.485	0.000	0.127	0.236	0.734
Symptoms	–	4.745	1	0.059	0.214	0.029	0.234	0.029	0.117	0.005	0.463

The independence of MBT/BST/Symptoms from HBT was tested with Pearson’s Chi-Squared Test or Fisher’s Exact Test. In LM, Fisher's exact test was used because of the small sample size. Phi (φ) was used for finding an association of MBT/BST/Symptoms with HBT and Cohens’ Kappa (κ) was calculated for checking the reliability of MBT/BST/Symptoms for diagnosing fructose or lactose malabsorption.

*HBT* hydrogen breath test. *MBT* methane breath test. BST: blood sugar test. *SE* standard error. *95% CI_low/CI_high* Cohens’ Kappa (κ) 95% confidence interval lower/upper threshold in ppm (MBT)/mmol/L (BST). *P* values < 0.05 were considered statistically significant. *df* degree of freedom.

### Blood sugar tests

Blood sugar test have been performed in parallel to all 269 breath tests. Using the recommended blood glucose cut-off of 1.1 mmol/L for diagnosing FM resulted in a sensitivity/specificity of 52%/32%, whereas for LM it resulted in a sensitivity/specificity of 50%/94%. The PPV/NPV for diagnosing FM based on BST was 54%/31% and for LM 87%/89%, respectively.

In FM the observed association between BST and HBT (Pearson Chi Square: Χ^2^(1) = 4.3, p = 0.037) showed a weak relationship (Phi coefficient: φ = − 0.152, p = 0.037). A degree of agreement cannot be defined, given negative Kappa values. BST and HBT in LM showed a significant association (Fisher’s exact test; p < 0.001) and a medium strength of relationship (Phi coefficient: φ = 0.493, p < 0.001). There was a moderate level of agreement between the two tests (Cohen’s κ = 0.485; 95% CI 0.236–0.734; p < 0.001)^30,31^.

### ROC curve analyses

**Figure 1:**
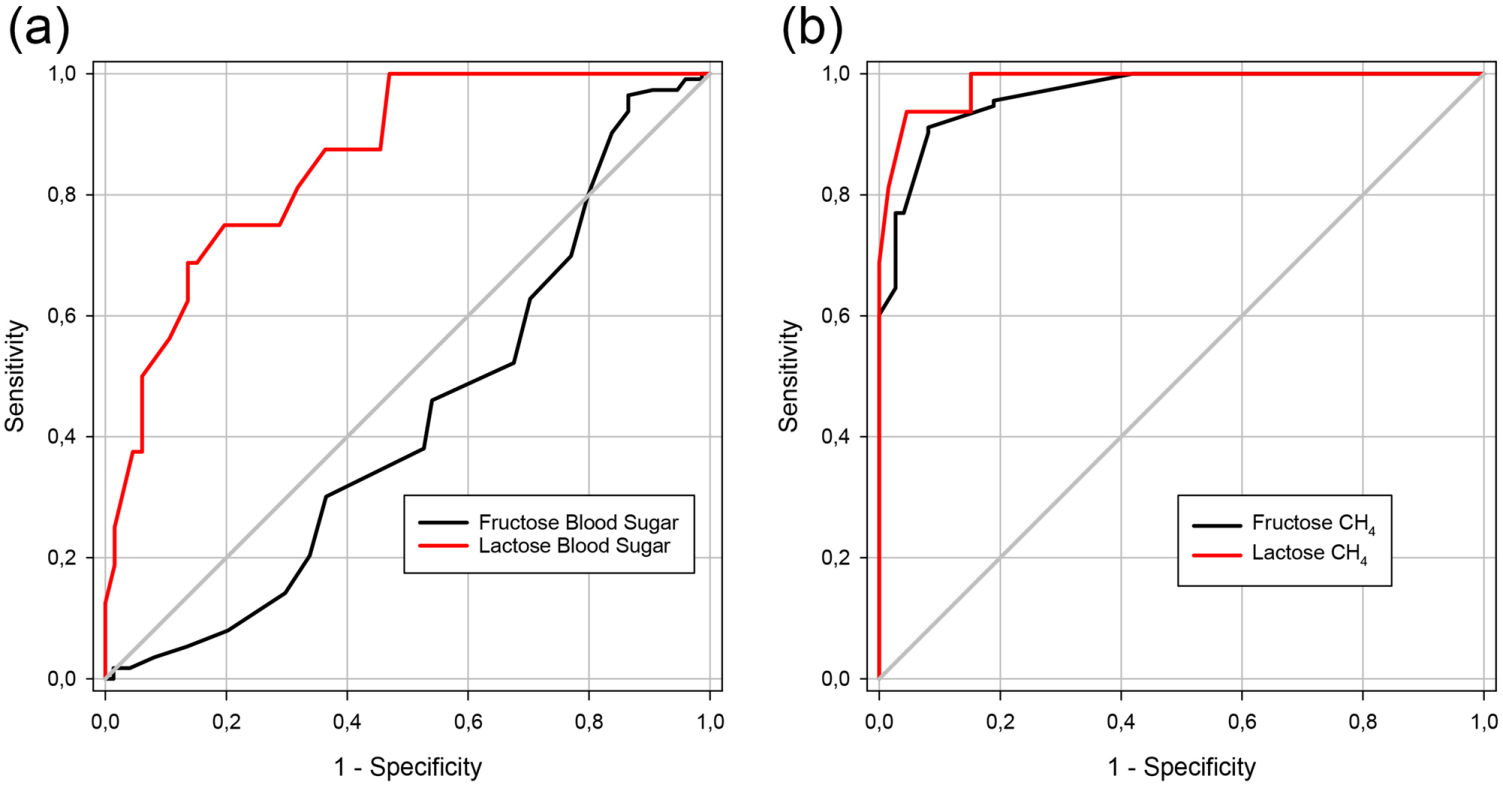
ROC curve analysis for blood sugar tests (**a**) and methane breath tests (**b**) in the diagnosis of fructose/lactose malabsorption. (**a**) Blood sugar was measured before administering the test sugar solution at baseline (0 min) and at 15/30/45/60 min and delta values were calculated. The highest blood sugar delta value was used and compared with the results of the corresponding hydrogen breath test (true positive). (**b**) ROC curve analysis for methane (CH_4_) breath tests in patients with suspected fructose and/or lactose malabsorption. CH_4_ was measured before administering the test sugar solution at baseline (0 min) and at 30/60/90/120 /150/180 min. The highest CH_4_ value was used and compared with the results of the corresponding hydrogen breath test (true positive).

A patient with a positive HBT was considered having FM/LM. For subsequent ROC curve analyses the highest CH_4_ value during the test (measured 0–180 min, Table 1) and the highest blood sugar delta value during the test, which is the maximum increase above baseline (measured at 15–60 min, Table 1) was used. Results of the ROC curve analyses are presented in Table 6 and in Fig. 1a,b.

**Table 6:** Accuracy of methane breath tests and blood sugar tests in carbohydrate malabsorption.

	AUC	SE	95% CI		P	Cut-off	Sensitivity	Specificity
**Fructose**								
MBT	0.967	0.011	0.946	0.989	0.000	6.25	91%	92%
BST	0.432	0.045	0.345	0.520	0.118	1.95	97%	14%
**Lactose**								
MBT	0.986	0.011	0.964	1.000	0.000	7.50	94%	96%
BST	0.857	0.047	0.765	0.949	0.000	1.75	75%	80%

*ROC* curve calculations for fructose/lactose malabsorption. *MBT* methane breath test. *BST* blood sugar test. *AUC* Area under the receiver operating characteristics (ROC) curve. *SE* Standard error. *95% CI* 95% confidence interval. *P* values < 0.05 were considered statistically significant. *Cut-off* values are given as ppm for MBT and mmol/L for BST.

MBT in suspected FM had an area under the ROC curve (AUROCC) of 0.967 (SE 0.011, 95% CI 0.946–0.989, p < 0.001) whilst BST resulted in an AUROCC of 0.432 (SE: 0.045, 95% CI 0.345–0.52, p = 0.118). In our study cohort the statistically optimal CH_4_ cut-off value for diagnosing FM was 6.25 ppm with a sensitivity of 91% and a specificity of 92%, whereas for BST it was 1.95 mmol/L (sensitivity 97%, specificity 13%).

MBT in patients with suspected LM had an AUROCC of 0.986 (SE: 0.011, 95% CI 0.964–1.000, p < 0.001). BST in patients with suspected LM resulted in an AUROCC of 0.857 (SE: 0.047, 95% CI 0.765–0.949, p < 0.001). The statistically optimal CH_4_ cut-off for detecting LM was 7.5 ppm (sensitivity 94%, specificity 96%) and for BST 1.75 mmol/L (sensitivity 75%, specificity 81%).

### Symptoms

Gastrointestinal symptoms during breath tests were reported in 51 (27%)/23 (28%) patients tested for FM/LM (Table 7). The remaining patients did not complain about any symptoms during the breath tests. The majority of children with symptoms during the breath test experienced abdominal pain (fructose breath test: 39 (21%), lactose breath test: 18 (22%)) whereas none of the children complained about flatulence or vomiting. 31 (17%)/8 (10%) children had a positive HBT for FM/LM and showed simultaneously symptoms. In FM/LM 11 (6%)/8 (10%) patients had more than one symptom during the test. Onset of gastrointestinal symptoms during fructose/lactose breath tests had a sensitivity of 27%/50%, a specificity of 73%/77%, a PPV of 61%/35% and a NPV of 40%/86% for LM/FM respectively. Cohen's κ indicated in FM no statistical agreement between symptoms and HBT (κ = 0.004; 95% CI − 0.110 to 0.118; p = 0.951) whereas in LM, Cohen's κ showed a weak level of agreement (κ = 0.234; 95% CI 0.005–0.463; p = 0.029). Symptoms which occurred prior to the test were not analyzed in this study.

**Table 7:** Gastrointestinal symptoms during breath tests in suspected fructose/lactose malabsorption.

	Fructose (n = 187)			Lactose (n = 82)		
**Patients, n (%)**						
Symptoms	51 (27)			23 (28)		
Symptom-free	136 (73)			59 (72)		

	Total	HBT positive	HBT negative	Total	HBT positive	HBT negative
Abdominal pain, n (%)	39 (21)	24 (13)	15 (8)	18 (22)	6 (7)	12 (15)
Bloating, n (%)	7 (4)	5 (3)	2 (1)	2 (2)	2 (2)	0 (0)
Nausea, n (%)	13 (7)	8 (4)	5 (3)	11 (13)	2 (2)	9 (11)
Flatulence, n (%)	0 (0)	0 (0)	0 (0)	0 (0)	0 (0)	0 (0)
Vomiting, n (%)	0 (0)	0 (0)	0 (0)	0 (0)	0 (0)	0 (0)
Diarrhea, n (%)	6 (4)	5 (3)	1 (1)	0 (0)	0 (0)	0 (0)
> 1 symptom, n (%)	11 (6)	9 (5)	2 (2)	8 (10)	2 (2)	6 (7)

## Discussion

We performed a large retrospective single-center study, which allowed us to analyze and compare the diagnostic value of HBT, MBT and BST in pediatric patients with chronic abdominal pain and suspected FM/LM. The aim of our study was to characterize the diagnostic accuracy of MBT and BST for diagnosing FM/LM. To date there is a lack of an international agreement on methodological aspects of breath tests in pediatric patients with suspected FM/LM. Published data on BST in this context show either its inferiority to HBT or is inconclusive^2,25,28,32–34^. Doses used for lactose breath testing ranges from 0.5 to 2 g/kg bodyweight up to a maximal dose of 25–50 g lactose^10,18,35^. In our cohort 2 g lactose per kilogram bodyweight (max. 50 g) was used and dissolved in water (20% solution). For fructose breath tests we used 1 g fructose per kilogram bodyweight (max. 25 g) dissolved in water (10% solution) which is in agreement with the current NACC recommendations^1,3,36,37^.

For the purpose of this study, patients having a baseline breath CH_4_ > 3 ppm were defined as CH_4_ producers. Accordingly, 64 (34%)/25 (31%) of our patients with suspected FM/LM were classified as CH_4_ producers. In other studies, the rate of CH_4_ producers in healthy subjects varies from 30 to 35%^22–24^.

Out of 187 patients tested for FM, 113 (60%) had a positive HBT result indicating FM. Simultaneous MBT in these patients had a low sensitivity (58%) but high specificity (100%) for diagnosing FM. The PPV of MBT equals 100%, indicating that all of the patients marked as positive for FM are actually affected by FM. A NHP is a patient suffering from FM even though the HBT is negative. In order to detect NHP in our cohort tested for FM, we performed MBT. Using the current HBT/MBT cut-off values for FM, we are not able to detect any NHP in our cohort since we did not identify any false positive MBT result. The benefit of measuring CH_4_ during fructose breath testing might, therefore, be limited. As expected, lowering the CH_4_ cut-off increased the sensitivity and reduced the specificity of MBT. Our ROC curve analyses showed an optimal CH_4_ cut-off value for detecting FM of 6.5 ppm. Using this cut-off value, we could increase the MBT sensitivity/specificity to 91%/92%. Lowering the CH_4_ cut-off to 5 ppm, we observed 29 of 74 (39%) false positive MBT where patients had a positive MBT and a negative HBT tested for FM. Therefore, it might be possible to detect NHP using a lower CH_4_ cut-off value. This is in agreement with an audit that suggested that identifying carbohydrate malabsorption by an increase of ≥ 20 ppm in methane producers needs to be questioned due to the variability in readings throughout testing. Instead, using a cut-off value of ≥ 5 ppm of methane on a single time point breath test seemed to identify methane producers^38^.

**Figure 2:**
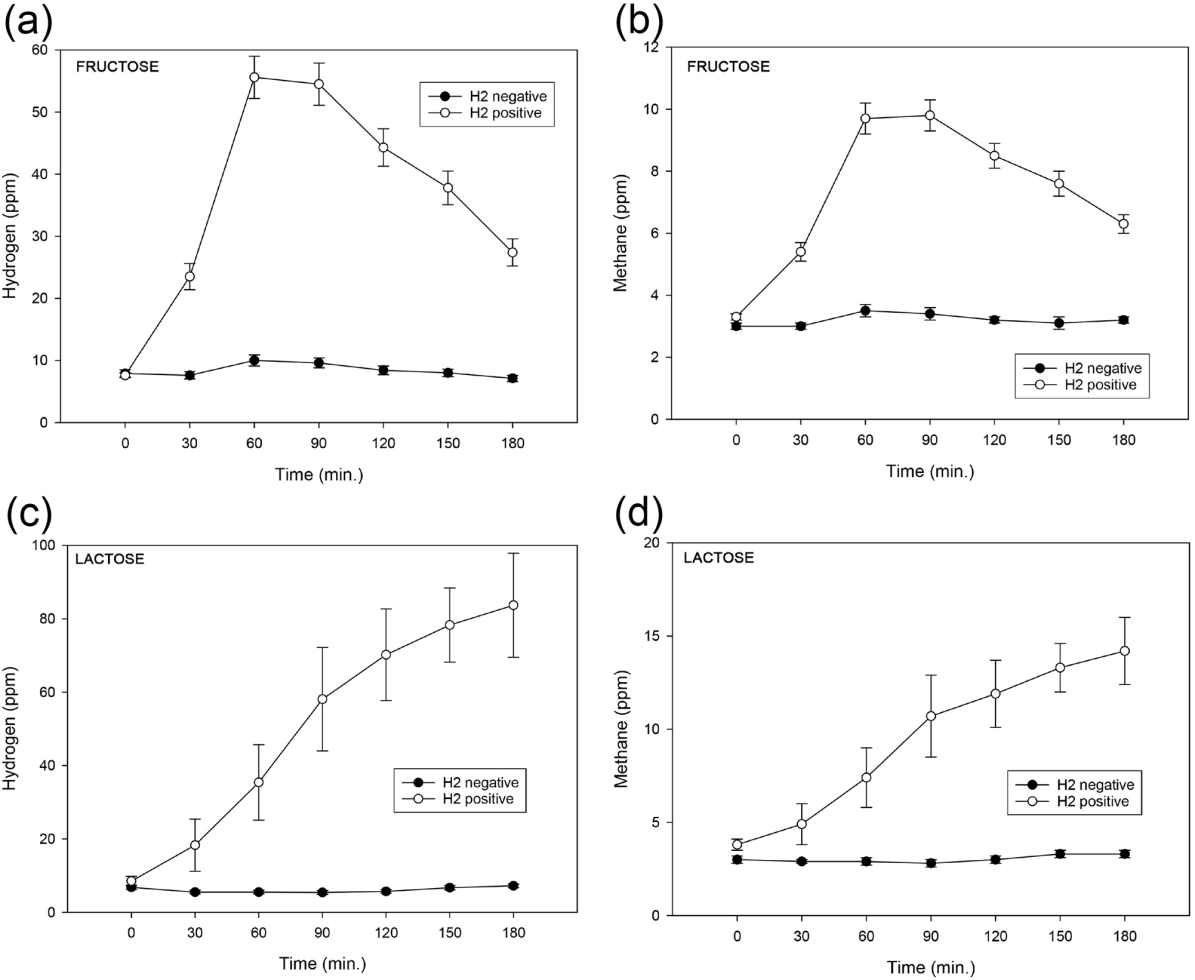
Time course analysis of endexpiratory hydrogen (H_2_) and methane (CH_4_) concentrations during hydrogen/methane breath tests (HBT/MBT) performed in children with suspected carbohydrate malabsorption. Shown are the hydrogen (**a**,**c**) and methane (**b**,**d**) concentrations during breath tests performed with fructose (**a**,**b**) and lactose (**c**,**d**). A positive HBT (H_2_ positive) was defined by an increase of H_2_ ≥ 20 ppm at 30/60/90/120 /150/180 min after administering of the respective test sugar solution. A CH_4_ concentration ≥ 10 ppm at any measurement was considered as a positive MBT (CH_4_ positive). Patients with an elevated baseline value (0 min: H_2_ > 20 ppm/CH_4_ ≥ 10 ppm) were excluded. Error bars at any given time are showing the standard error of the mean.

As shown in Fig. 2a,b, time course analysis showed that changes in endexpiratory H_2_ and CH_4_ concentrations during fructose breath tests have a similar pattern. They increase during the test until they reach a peak at 60 to 90 min following by a prompt decrease. Similar results were reported by Rao et al.^22^. This indicates, that the production of H_2_ and CH_4_ during breath tests behaves similar and a test duration of 3 h is sufficient. During HBT and MBT in patients with suspected LM we found a different pattern. H_2_ and CH_4_ values seem to constantly rise without reaching a peak within our test duration (Fig. 2c,d). A longer test duration has been discussed by other authors^27^. The different patterns in FM and LM might be caused by the different metabolic pathways of fructose/lactose.

**Figure 3:**
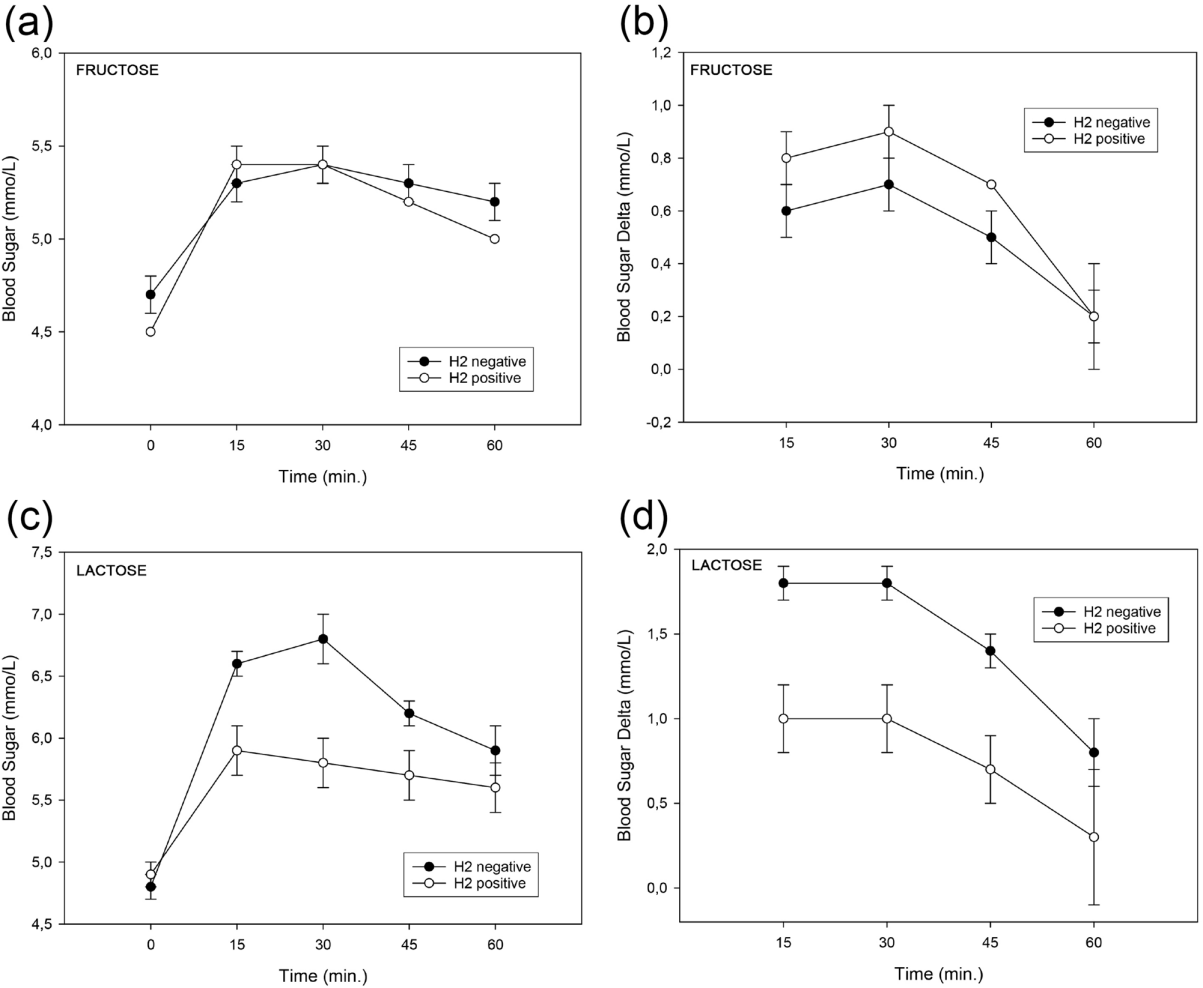
Parallel blood sugar testing and hydrogen breath test (HBT) in children with suspected carbohydrate malabsorption. Time course analysis of blood sugar levels in children with suspected fructose (**a**,**b**) and lactose (**c**,**d**) malabsorption. A positive HBT (H_2_ positive) was defined by an increase of H_2_ ≥ 20 ppm at 30/60/90/120 /150/180 min after administering fructose/lactose. Patients with an elevated H_2_/CH_4_ baseline value (0 min: H_2_ > 20 ppm/CH_4_ ≥ 10 ppm) were excluded. Blood sugar levels were measured before administering the test sugar solution (0 min, “baseline”) and at 15/30/45/60 min. Shown are the total blood sugar values (**a**,**c**) or the blood sugar delta values (**b**,**d**). Error bars indicate the standard error of the mean.

BST have a statistically significant but only weak connection to HBT. ROC curve analyses showed, that BST are not sufficient to diagnose FM even when using a statistically optimal cut-off value of 1.95 mmol/L. Interestingly, the blood sugar values increase with a peak at either minute 15 or 30 in both groups (HBT positive and negative), as shown in Fig. 3a. This could be partly an effect of stress followed by the release of glucocorticoids^39^. An increase of blood glucose < 1.1 mmol/L for all BST was considered positive^2,28^. Blood sugar delta values are in both groups (HBT positive and negative) mostly below the cut-off (Fig. 3b), whereas patients having FM showed higher delta values. Therefore, we conclude that BST has no additional value in diagnosing FM in children.

HBT results may not correlate with gastrointestinal symptoms in FM. Only 27% of our patients (HBT positive and negative) complained about symptoms after oral application of fructose. Since the sensitivity (27%) and the specificity (73%) of symptoms during HBT for diagnosing FM is low and Cohens’ κ showed no level of agreement, symptoms are not a reliable criterion to identify healthy or affected children. Using symptoms during breath test for diagnosing FM showed a higher rate of false negative patients (82 of 113 patients; 73%) compared to 25% of subjects in the cohort of adult patients Rao et al. used^22^. Studies showed that adult and pediatric individuals might have gastrointestinal symptoms during breath tests although the HBT is negative^1,22^. These symptoms may occur due to a hyperosmolar effect, assuming an incomplete small intestine absorption of the carbohydrate. In our cohort of patients with suspected FM, this effect was observed in 20 patients (11%). In a recent study by Helwig et al.^40^, diarrhea and bloating significantly correlated with the total H_2_ maximum, the maximum H_2_ increase and the AUROCC of the HBT with fructose. As indicated by these findings, symptoms which occur during breath tests are not a reliable parameter to diagnose FM. Given the reported lack of association of the induction of symptoms (carbohydrate intolerance) with a positive fructose breath test result (carbohydrate malabsorption) we would recommend a diagnostic dietary intervention even in a highly symptomatic patient with a negative breath test result. However, we would not necessarily recommend a dietary intervention in a patient with a positive breath test but no symptoms. Furthermore, the issue of breath test results and poor symptom correlation needs further improvement given the poor reproducibility and low predictive value for symptom responses for example in fructose breath testing for clinical application in functional GI diseases^7^. In addition, an insufficient hydrogen response during a breath test in patients with suspected carbohydrate malabsorption might be caused by efficient hydrogen utilization by methanogens, sulphate-reducing bacteria and/or acetogens. Lactulose cannot be absorbed in the small intestine and is fermented in the large intestine. Therefore, a patient’s ability to produce hydrogen and/or methane can be assessed through a lactulose breath test, which provides a positive control for further breath testing in patients with suspected fructose/lactose malabsorption. Therefore, lactulose breath tests are frequently used as a baseline test in clinical practice to improve the interpretation of subsequent breath test results. Although HBT with lactulose cannot be routinely recommended to identify non-hydrogen producers, such a baseline test could enable a more rational interpretation of the results after tests with other sugars by specifying the strength of hydrogen production in the patient. It has been suggested that a lactulose HBT allows an optimal duration of the subsequent breath tests and provides possible mechanistic information on carbohydrates malabsorption^41^. However, lactulose can alter transit time which may not occur in the same way for fructose or lactose. Hence the time of rise of breath hydrogen or methane may not be consistent for different sugars in the same individual.

Following the present test parameters published by the NACC, HBT for LM were positive in 16 (20%) of our patients with suspected LM. 25 (31%) children tested for LM were CH_4_ producers but at the same time only 14 (17%) had a sufficient increase in CH_4_ concentration (CH_4_ ≥ 10 ppm over baseline, therefore are CH_4_ positive) to successfully be diagnosed with LM. The values of endexpiratory H_2_ and CH_4_ concentrations at different measurement time points during breath tests in children with suspected LM showed different patterns compared to FM. H_2_ and CH_4_ values seem to constantly rise without having a peak during the test duration of 3 h (Fig. 2c,d). MBT showed a good sensitivity (81%) and an excellent specificity (99%). The reported number of NHP varies widely from 2 to 43%^4^. Taken together, these findings suggest that it is difficult to identify NHP with the current criteria suggested by the NACC. The detailed mechanism of production and exhalation of CH_4_ remains unclear as well as the importance of identifying NMP^42^. Statistically, there is a complete agreement between HBT and MBT in our study. Our ROC curve analyses revealed an excellent AUROCC (0.986) and an optimal methane cut-off at 7.5 ppm (sensitivity 94%, specificity 96%) for diagnosing LM. Tormo et al. stated that the pattern of CH_4_ excretion is independent of H_2_^42^. Our tests revealed that H_2_ and CH_4_ behave similar in most tests, with only 1 patient tested for LM having a positive MBT whilst having a negative HBT. If we lower the CH_4_ cut-off to 5 ppm during testing for LM, we observe 17 of 66 (26%) false positive MBT results where patients had a positive MBT and a negative HBT. Thus, it might be possible to detect NHP using a lower CH_4_ cut-off value.

After lactose ingestion 12 (15%) patients had a positive BST. Of those 12 children, only 8 had a positive HBT and thus were correctly diagnosed with LM based on BST. A low sensitivity (50%) but a high specificity (94%) was found in BST for diagnosing LM. Consistent with our results, Ruzsanyi et al. showed that a better agreement of HBT with BST was found in patients with a negative BST and a negative HBT compared with patients having positive results in both HBT and BST^28^. Figure 3a,b shows an early increase in blood sugar levels followed by a decrease during breath tests in patients with FM. Figure 3c,d reveals, that children without LM had a higher increase in blood sugar levels during breath tests than those having LM. In agreement with that, the blood sugar delta values are lower during breath test in the group having LM (Fig. 3d).

Of all tested patients, 23 (28%) showed at least one gastrointestinal symptom after lactose ingestion while 59 (72%) did not complain about any symptoms. Only 8 patients out of 16 patients with positive HBT using lactose reported gastrointestinal symptoms during the breath test. There is only a poor and insignificant correlation of symptoms and HBT in LM and the level of agreement is weak. A low sensitivity (50%) and moderate specificity (77%) of symptoms indicate that symptoms reported during the HBT are not a reliable parameter for diagnosing LM which is in accordance with other studies^2,28^.

Due to the fact that our study cohort was recruited between 01/2012 and 05/2015 and therefore before the NACC recommendations were updated in 2017^27^ we used max. 50 g lactose instead of max. 25 g for breath tests. High doses of lactose showed a significant increase of H_2_ production in patients with other gastrointestinal diseases^25^. We are aware that these high lactose doses also could have influenced our results and might be a potential limitation of our study. It has been shown that 25 g of fructose can be absorbed by healthy individuals^22^ and is the recommended by the NACC as the max. dose for breath tests investigating FM. However, there is evidence that the capacity to absorb fructose can be easily overwhelmed by administering an excessive amount of fructose^22^.

## Conclusions

Gastrointestinal symptoms after fructose/lactose intake (carbohydrate intolerance) and carbohydrate malabsorption are a common problem in children. Due to the lack of diagnostic standardization and contradictory results of available tests, it is difficult to establish a reliable and correct diagnose. With the currently used cut-offs for breath tests in FM, additional MBT and BST did not show any further diagnostic advantage. In addition, repeated BST in children is an invasive approach in order to identify FM. Using current cut-off values for CH_4_, it was not possible to identify NHP in patients with suspected FM, since we did not identify false positive MBT. In LM, MBT can identify most of the patients with LM, also with a good test specificity. BST can correctly identify individuals with normal carbohydrate absorption and BST might therefore be an option in patients who cannot participate in a breath test for suspected LM. For FM and LM, reported symptoms during the breath test are not a reliable method to identify children with carbohydrate malabsorption but might be a measure for carbohydrate intolerance. If the currently recommended cut-off values for MBT are lowered, it might be possible to identify NHP, a group of patients, which were formerly not recognized. The role of patients producing only small amounts of CH_4_ remains unclear. As NHP in HBT, an existence of NMP in MBT should be considered and further investigated. Overall, a combined test approach might help in diagnosing children with carbohydrate intolerance/malabsorption.
